# Maternal Separation Induced Visceral Hypersensitivity Evaluated via Novel and Small Size Distention Balloon in Post-weaning Mice

**DOI:** 10.3389/fnins.2021.803957

**Published:** 2022-01-28

**Authors:** Enfu Tao, Gao Long, Ting Yang, Bo Chen, Rui Guo, Diya Ye, Marong Fang, Mizu Jiang

**Affiliations:** ^1^Endoscopy Center and Gastrointestinal Laboratory, Children’s Hospital, Zhejiang University School of Medicine, National Clinical Research Center for Child Health, National Children’s Regional Medical Center, Hangzhou, China; ^2^Department of Pediatrics, Wenling Maternal and Child Health Care Hospital, Wenling, China; ^3^Institute of Neuroscience and Gastrointestinal Laboratory, Children’s Hospital, Zhejiang University School of Medicine, Hangzhou, China

**Keywords:** maternal separation, visceral hypersensitivity, distention balloon, irritable bowel syndrome, mice

## Abstract

Early life stress (ELS) disposes to functional gastrointestinal diseases in adult, such as irritable bowel syndrome (IBS). Maternal separation (MS) is a well-known animal model of IBS and has been shown to induce visceral hypersensitivity in adult rats and mice. However, to the best of our knowledge, it has not been reported whether MS induces visceral hypersensitivity in young mice, such as the post-weaning mice. Moreover, the method for evaluation of visceral sensitivity also has not been described. Accordingly, the present study aims to evaluate the visceral sensitivity caused by MS in post-weaning mice and develop a novel and small size distention balloon for assessment of visceral sensitivity of such mice. Male pups of C57BL/6 mice were randomly divided into two groups, MS (*n* = 12) and non-separation (NS) (*n* = 10). MS pups were separated from the dams through postnatal days (PND) 2 to 14, while NS pups were undisturbed. After, all pups stayed with respective dams and were weaned at PND 22. Visceral sensitivity was evaluated by colorectal distention (CRD) with a novel and small size distention balloon at PND 25. The threshold of abdominal withdrawal reflex (AWR) scores were significantly lower in MS than NS. In addition, AWR scores at different pressures of CRD were significantly higher in MS than NS. The results demonstrate that MS induced visceral hypersensitivity in post-weaning mice. The designed small size distention balloon for evaluation of visceral sensitivity is of significance to further study the pathophysiology of IBS from early life to adulthood.

## Introduction

Irritable bowel syndrome (IBS) is a functional gastrointestinal disorder and newly called “disorders of the gut–brain interaction” based on Rome IV classification (Drossman, 2016). Although the mechanism underlying the pathogenesis of IBS is not well understood, brain–gut axis dysfunction and visceral hypersensitivity are two of the main characteristics of IBS, which may relate to psychological distress (anxiety, depression, impulsiveness, anger) in children (Iovino et al., 2009). A large number of studies have shown that early life stress (ELS) can result in persistent changes in the central stress response systems, visceral hypersensitivity, and increase predisposition to IBS (Burke et al., 2017; Ju et al., 2020; Rincel and Darnaudéry, 2020).

Maternal separation (MS), which mimics the ELS, is a well-known animal model of IBS, and it has been shown to induce visceral hypersensitivity in adult rats (Botschuijver et al., 2019; Fan et al., 2020) and mice (Riba et al., 2017, 2018). Furthermore, MS disrupts intestinal homeostasis (Wong et al., 2019), activates the immune system (Barreau et al., 2008; O’Malley et al., 2013), alters gut microbiota (O’Mahony et al., 2009), and induces lasting alterations in neuronal circuits, neurotransmitter systems, neuronal architecture, and plasticity that are further associated with emotional and cognitive information processing (Caliskan et al., 2020). These alterations derived from MS are based on the fact that the perinatal period is a crucial window for the intestinal barrier, the immune system, and the gut microbiota to mature to establish an appropriate complex relationship (Renz et al., 2011). However, the precise and dynamic process of the changes that underlie the development of IBS from MS pups to adults is still poorly understood. Whether ELS will induce IBS in early life, such as childhood, is not elucidated. Accordingly, it is of significance to evaluate IBS-like phenotype including visceral hypersensitivity caused by MS in young mice, such as the period after weaning.

Colorectal distention (CRD) is a widely used method for assessing visceral hypersensitivity (Larsson et al., 2003; Christianson and Gebhart, 2007; Annahazi et al., 2009; Huang et al., 2021). However, CRD for evaluation of visceral sensitivity has not been reported in post-weaning mice. One of the main reasons may be that there is no suitable distention balloon for CRD of such mice. Therefore, it is urgent to develop a tool for CRD in post-weaning mice.

Accordingly, the present study aims to evaluate the visceral sensitivity caused by MS in post-weaning mice and develop a novel and small size distention balloon for assessment of visceral sensitivity of such mice.

## Materials and Methods

### Animal Preparation

**FIGURE 1:**
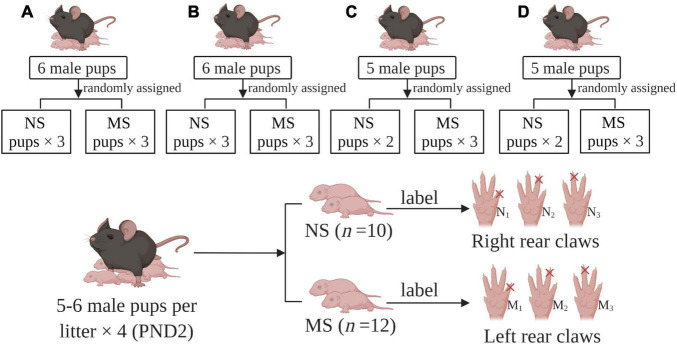
Group assigned and labels. **(A–D)** gestational mice delivering on the same day were included in the experiments. PND: postnatal day, MS: maternal separation, NS: non-separation, M_1–3_: labels for maternal separation pups with one of the left rear claws cut off, N_1–3_: labels for maternal separation pups with one of the right rear claws cut off.

Specific pathogen-free (SPF) C57BL/6 mice were used in the experiment. Protocols for animal research were approved by the Zhejiang University Ethics Committee for Animal Research. C57BL/6 male and female mice (8 weeks old) were bred for mating at the ratio of 1:2. They were housed (cage size 318 × 202 × 135 mm) and maintained on a 12-h light–dark cycle (light turned on at 9:00 am and turned off at 9:00 pm automatically) with access to food and water *ad libitum*, at a room temperature of 25–26°C, under a relative humidity of 50 ± 5%. Ventilation rate reached 8–15 times/h and the ammonia concentration was less than 20 ppm. Mice received irradiated standard chow and wood chip bedding. The bedding was changed twice weekly. After mating, gestational mice were housed individually. Gestational mice delivering on the same day were included in the experiments. To avoid the effects of stress on dams, litters were not disturbed at the first day after delivery. On postnatal day (PND) 2, four litters were standardized to five to six male pups per litter. Female pups were killed and not used for following experiments to avoid female hormonal cycle interference. Then male neonatal mice were randomly assigned to the MS group (*n* = 12) and non-separation (NS) group (*n* = 10). During grouping, all male pups were labeled by cutting off one of the rear claws, with left for MS, and right for NS. The procedure of animal preparation is summarized in Figure 1.

### Maternal Separation Paradigms

Maternal separation was performed as previously described (Riba et al., 2018; Wong et al., 2019) with some modifications. Briefly, on PND 2–14, MS pups were removed from dams daily for 3 h consecutively (9:00 am to 12:00 am) and were individually placed into a plastic box (size 180 × 130 × 55 mm) with the wood chip bedding in a separated room with ambient temperature 26 ± 2°C. The bedding was changed once weekly. After separation, MS pups were returned to dams and left undisturbed, whereas NS pups were nursed as usual. All pups were weaned on PND 22. After that, five to six per cage of MS and NS mice were housed (cage size 318 × 202 × 135 mm) independently.

### Body Weight Measurements

Both MS and NS mice were measured for body weight daily by electronic scale from PND 2 to 25 before separation, and body weight was recorded in a pre-made form.

### Novel and Small Size Distention Balloon

**FIGURE 2:**
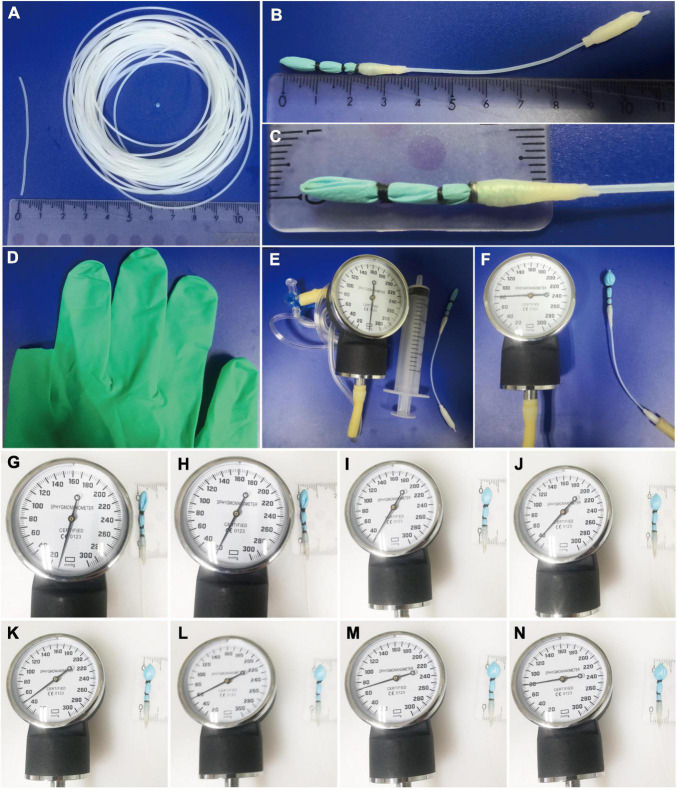
Novel and small size distention balloon. **(A)** Teflon tube with 1 mm outer diameter, 0.6 mm internal diameter. **(B–D)** The distention balloon was made from a little finger of a small size latex glove (2 cm in length) placed into Teflon tubes and were tied with 6-0 silk thread at a distance of 1, 1.5, and 2 cm, respectively, from the tip of the glove finger. The balloon was about 3 mm in diameter, 1 cm long, and was subsequently sealed using parafilm in a manner of winding at a distance of 2 cm from the tip of the glove finger. **(E)** Pressure gauge, 10-ml syringes, and distention balloon (from left to right). **(F)** Pressure gauge shows the distention balloon working at a pressure of 80 mmHg. **(G–N)** Sizes of distention balloon at 10–80 mmHg pressure.

The distention balloon was made from a little finger of a small size latex glove (2 cm in length) placed into Teflon tubes (10 cm length; outer diameter, 1 mm; internal diameter, 0.6 mm) purchased from Taobao, and were tied with 6-0 silk thread at a distance of 1, 1.5, and 2 cm, respectively, from the tip of glove finger. Before the latex glove was placed into Teflon tubes, at the front of the Teflon tube, two side holes were sheared with ophthalmic scissors at an oblique angle on both sides of the tube to increase the uniformity of balloon inflation. This step was critical in that it helped avoidance of glove tip obstructing the tube. The balloon was about 3 mm in diameter, 1 cm long, and was subsequently sealed using parafilm in a manner of winding at a distance of 2 cm from the tip of the glove finger. The end of the Teflon tube was also wrapped by parafilm to increase the diameter size to connect with a three-way tube. The two ends of the tube connected 10-ml syringes and pressure gauge, respectively. In this way, the pressure of the distention balloon could be monitored in real time (Figure 2).

### Abdominal Withdrawal Reflex

Abdominal withdrawal reflex score was evaluated by colorectal distention (CRD) on mice between 10:00 am and 4:00 pm at PND 25 according to the previous study with some modifications (Al-Chaer et al., 2000; Yu et al., 2012; Zhang et al., 2020). Briefly, mice were allowed free access to food and water before the experiment. Anuses were lubricated with Vaseline ahead of the experiment. The distention balloon was introduced into the rectum at 1.0 cm from the anus and fixed at the base of the tail under 1.5% isoflurane (RWD Life Science Co., Ltd. Shenzhen, China) anesthesia. Then, mice were placed in a hollow cylinder made from transparent acrylic (cylinder size 6 cm long, 3 cm inner diameter, purchased from Taobao) and allowed to recover 30 min fully from the anesthesia and adaption of the balloon. One end of the cylinder was fixed and the other was a lid (3 cm diameter). There was a hole (1.5 cm diameter) in the middle of both ends. The hole in the fixed end was used for ventilation, while the hole in the lid was used for pulling out the tail and then to connect with a three-way tube. In this way, the mice could not move backward and forward but could move up and down, such as lifting of abdomen, body arching, and lifting of pelvic structures, and remained in a relative quiescent state. All the mice were evaluated with the same size balloon.

**FIGURE 3:**
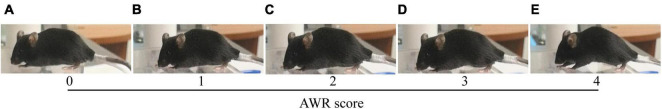
AWR score based on mice behavioral response to CRD. **(A)** 0, no behavioral response to CRD; **(B)** 1, brief head movement followed by immobility; **(C)** 2, contraction of abdominal muscles; **(D)** 3, lifting of abdomen; **(E)** 4, body arching and lifting of pelvic structures (Gosselin et al., 2010). AWR, abdominal withdrawal reflex; CRD, colorectal distention.

The AWR scores were graded on a scale of 0–4 (Figure 3): 0, no behavioral response to CRD; 1, brief head movement followed by immobility; 2, contraction of abdominal muscles; 3, lifting of abdomen; 4, body arching and lifting of pelvic structures (Gosselin et al., 2010). For measuring the pressure threshold of AWR scores 1, 2, 3, and 4, the colorectal balloon was progressively inflated, from 0 mmHg of pressure to the maximum pressure, and different degrees of pain behaviors (0–4) displayed and the real-time pressure was recorded in a pre-made form. When recording, inflation of the balloon was ceased but the pressure was retained. In this manner, the pressure threshold of AWR scores 1, 2, 3, and 4 was determined and recorded continuously. After 5 min of rest, the repeated measurement was performed. Then the mice had 5 min of rest for measuring the AWR. For measuring the AWR at different pressures of the balloon, the balloon was rapidly inflated to constant pressure (10, 20, 30, 40, 50, 60, 70, and 80 mmHg). Each pressure was maintained for 5 s, and afterwards, the balloon was rapidly and completely deflated. After 20 s of rest, the next pressure was performed. In this way, AWR at the pressures of 10–80 mmHg was determined and recorded in sequence. Likewise, after 5 min of rest, the repeated measurement was performed. Pressures are always presented from lowest to highest. All measurements were carried out by two blinded observers. They were blinded to the experimental group (NS or MS group). The average value of the measurements (the pressure threshold of AWR scores 1, 2, 3, and 4 and AWR score at the pressure of 10, 20, 30, 40, 50, 60, 70, and 80 mmHg) was recorded, respectively.

### Statistical Analysis

The distribution of data was analyzed by Kolmogorov–Smirnov test. Data were shown to fit a normal distribution and were expressed as mean ± SD. The sample size was analyzed using Power Analysis and Sample Size (PASS 15.0). Differences between two groups were determined by two-way repeated measures ANOVA with Sidak’s multiple comparisons test. All data were analyzed by IBM Statistical Package for the Social Sciences (SPSS), version 23 (IBM SPSS Statistics). A *P*-value < 0.05 was considered statistically significant.

## Results

### Body Weights

**FIGURE 4:**
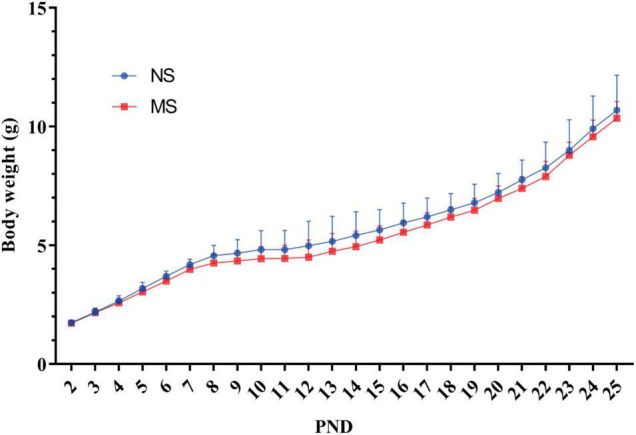
Comparison of mice body weight between MS and NS. There was no significant difference of mice body weight between MS and NS. NS, non-separation; MS, maternal separation; PND, postnatal day. MS, *n* = 12, NS, *n* = 10.

There was no significant difference of mice body weight between MS and NS [two-way repeated measures ANOVA (ELS × PND) with Sidak’s multiple comparisons test, no interaction: *F*(23,483) = 0.55, *P* = 0.96; also no significant main effect of ELS: *F*(1,21) = 1.73, *P* = 0.20; however, it had a significant main effect of PND: *F*(23,483) = 672.5, *P* < 0.0001] (Figure 4).

### Abdominal Withdrawal Reflex Threshold

**FIGURE 5:**
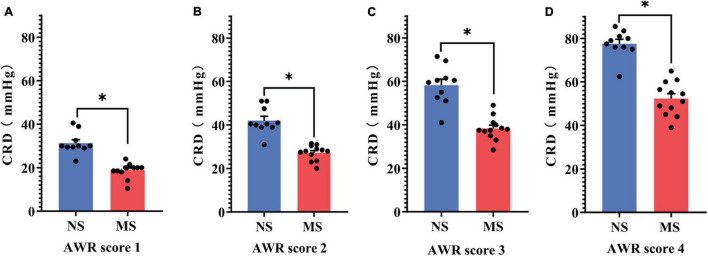
Comparison of the threshold of AWR score between MS and NS. **(A)** The CRD threshold of ARW score 1. **(B)** The CRD threshold of ARW score 2. **(C)** The CRD threshold of ARW score 3. **(D)** The CRD threshold of ARW score 4. CRD, colorectal distention; AWR, abdominal withdrawal reflex; MS, maternal separation; NS, non-separation, *P < 0.0001. MS, *n* = 12, NS, *n* = 10.

The CRD threshold of ARW scores 1 (18.83 ± 3.54 vs. 31.3 ± 5.07 mmHg, *P* < 0.0001) (Figure 5A), 2 (27.25 ± 3.49 vs. 42.05 ± 6.15 mmHg, *P* < 0.0001) (Figure 5B), 3 (38.33 ± 5.29 vs. 58.25 ± 8.93 mmHg, *P* < 0.0001) (Figure 5C), and 4 (52.33 ± 7.72 vs. 77.6 ± 6.29 mmHg, *P* < 0.0001) (Figure 5D) was significantly lower in MS compared with NS, respectively, [two-way repeated measures ANOVA (ELS × pressure) with Sidak’s multiple comparisons test, interaction: *F*(3,60) = 16.64, *P* < 0.0001; and it had significant main effect of ELS: *F*(1,20) = 63.80, *P* < 0.0001; also significant main effect of pressure: *F*(3,60) = 617, *P* < 0.0001].

### Abdominal Withdrawal Reflex Versus Pressure

**FIGURE 6:**
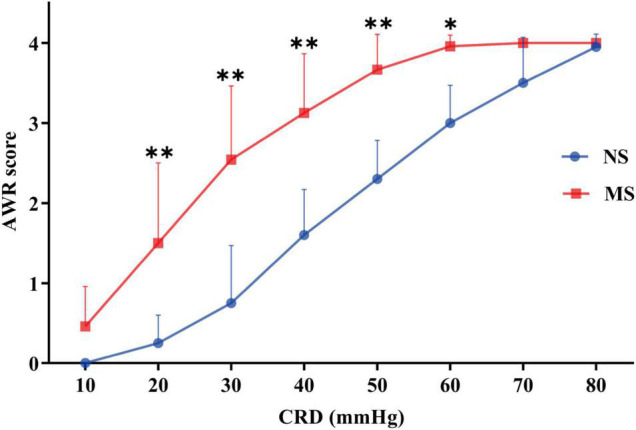
AWR score at different pressures of CRD in MS and NS. The ARW scores at 20, 30, 40, 50, and 60 mmHg were significantly higher in MS compared with NS, respectively. However, there was no difference of AWR score at 10, 70, and 80 mmHg pressure of CRD between MS and NS (*P* > 0.05). CRD, colorectal distention; AWR, abdominal withdrawal reflex; MS, maternal separation; NS, non-separation, *P < 0.001, ^**^P < 0.0001. MS, *n* = 12, NS, *n* = 10.

The ARW scores at 20 mmHg (1.50 ± 1.0 vs. 0.25 ± 0.35, *P* < 0.0001), 30 mmHg (2.54 ± 0.92 vs. 0.75 ± 0.72, *P* < 0.0001), 40 mmHg (3.13 ± 0.74 vs. 1.6 ± 0.57, *P* < 0.0001), 50 mmHg (3.67 ± 0.44 vs. 2.3 ± 0.15, *P* < 0.0001), and 60 mmHg (3.96 ± 0.14 vs. 3.0 ± 0.47, *P* = 0.0005) were significantly higher in MS compared with NS, respectively, [two-way repeated measures ANOVA (ELS × pressure) with Sidak’s multiple comparisons test, interaction: *F*(7,140) = 11.20, *P* < 0.0001; and it had significant main effect of ELS: *F*(1,20) = 36.85, *P* < 0.0001; also significant main effect of pressure: *F*(7,140) = 236.1, *P* < 0.0001]. However, there was no difference of AWR score at 10, 70, and 80 mmHg pressure of CRD between MS and NS (*P* > 0.05). These results are shown in Figure 6.

## Discussion

This study evaluated visceral hypersensitivity induced by MS in post-weaning mice and developed a novel and small size distention balloon for CRD to evaluate visceral sensitivity in such mice. To our knowledge, the study is the first time to report that MS induced visceral hypersensitivity in post-weaning mice and also is the first time to develop a novel distention balloon for CRD to evaluate visceral sensitivity in such mice.

Visceral hypersensitivity as one of the major characteristics of IBS had been widely studied in rats with the model of MS (Hyland et al., 2009; Gosselin et al., 2010; Enck et al., 2016; Botschuijver et al., 2017). However, it remains difficult to study this model to mice because MS led to only mild behavioral anomalies in adult mice (Millstein and Holmes, 2007). Moloney et al. (2012) developed a mouse model of MS stress-induced visceral hyperalgesia as measured by CRD (Moloney et al., 2012); from then on, MS of mice model were extensively conducted to study the mechanism underlying the pathogenesis of IBS, such as intestinal dysbiosis, intestinal dysfunction, intestinal permeability alteration, and IBS-like anxiety and depression behaviors (De Palma et al., 2015; Li et al., 2017; Riba et al., 2017; Rincel et al., 2019; Zhou et al., 2020). Indeed, compared with the rat model, the mouse model of MS had the advantages of its small size, relatively high reproductivity, inexpensive, and can be genetically manipulated (Kong et al., 2013; Shaqour et al., 2021). Because early postnatal life is a critical period during which both brain and gut undergo important maturation (Borre et al., 2014; Sharon et al., 2016), this period constitutes a critical window of sensitivity to stress (Rincel and Darnaudéry, 2020). Numerous studies had shown that exposure to adverse stress during childhood has been repeatedly associated with increased vulnerability to both psychiatric and gastrointestinal disorders such as IBS (Chitkara et al., 2008; Nemeroff, 2016). Moreover, a recent study focused on the effect of MS on young rodents and the process of the changes from early life to adulthood (Yi et al., 2017). Indeed, dynamic investigation of the period during early life to adult may, at least partly, contribute to exploring the underlying mechanism of pathogenesis of IBS. Accordingly, with the development of transgenic mice, optogenetic technology, tracing techniques, and so on (Ritsma et al., 2014; Boesmans et al., 2015; Chalazonitis et al., 2020; Liu et al., 2020), the novel distention balloon may be proved useful to explore the potential mechanism of pathogenesis of IBS from early life to adulthood in the future.

The underlying mechanism of MS-induced visceral hypersensitivity has been widely studied in adult rats and mice (Tang et al., 2017; Fan et al., 2020; Chen et al., 2021; Huang et al., 2021). It was reported that sensitization of corticotropin-releasing factor (CRF) neurons in the hypothalamic paraventricular nucleus (PVN) plays an important role in the pathogenesis of visceral pain (Zhang et al., 2016). A recent study suggested that *a priori*, PVN*^CRF^*-projecting GABAergic neurons in bed nucleus of stria terminalis (BNST)–anterior ventral (AV) region participated in the occurrence of visceral hypersensitivity induced by MS in adult mice, providing new insight into the neural circuit mechanism of chronic visceral pain. Moreover, MS induced the activation of PVN mast cells, which secreted numerous proinflammatory mediators that might involve in neighboring CRF neuronal activity, ultimately directly inducing visceral hypersensitivity in adulthood rats (Chen et al., 2021), implying central immuno-inflammatory process also taking part in the MS-induced visceral hypersensitivity. A recent study also using MS paradigm revealed that MS-induced visceral pain and visceral hypersensitivity were associated with the underfunction of small-conductance calcium-activated potassium channel subtype 2 (SK2) in the spinal dorsal horn (DH). The results showed that specific ion channel was also implicated in MS-induced visceral hypersensitivity. Furthermore, Piezo2 was reported as an important mechanogated ion channel that was involved in light touch sensitivity and inflammatory allodynia (Yang et al., 2016; Szczot et al., 2018). It was reported that in IBS patients, the expression of Piezo2 significantly correlated to the visceral sensitivity (Bai et al., 2017), indicating that Piezo2 was a candidate biomarker for visceral hypersensitivity in IBS. In rats, Piezo2 knock-down in dorsal root ganglion (DRG) attenuated visceral sensation to innocuous stimuli in control rats and both innocuous and noxious stimuli with neonatal irritation (Yang et al., 2016). These results implied the potential mechanism of Piezo2 in the visceral hypersensitivity of IBS. However, whether Piezo2 involve in MS-induced visceral hypersensitivity is not clear. Accordingly, it is worth of further study. In addition, some important signaling pathways also played a role in MS-induced visceral hypersensitivity. One study demonstrated that MS caused visceral hypersensitivity by activating brain-derived neurotrophic factor (BDNF)–tyrosine kinase receptor B (TrkB)–protein kinase Mζ (PKMζ) signaling in the thoracolumbar spinal cord of adult rats (Fan et al., 2020). In addition, sensitization and upregulation of microglial toll-like receptor 4 (TLR4) signaling activity in the PVN (Tang et al., 2017), ephrin-B2/EphB1 spinal signaling pathway (Theofanous et al., 2020), and nerve growth factor (NGF)–mediated tropomyosin receptor kinase A (TrkA) signaling (Wong et al., 2019) were involved in MS-induced visceral hypersensitivity. Moreover, various important signaling molecule dysfunctions were also involved in MS-induced visceral hypersensitivity, such as elevated expression of Nesfatin-1 in the dorsal raphe nucleus (DRN) (Zhang et al., 2018). Together, these studies focused the mechanism of MS-induced visceral hypersensitivity in adulthood. However, it remains unclear when these alterations are initial and whether these alterations occur earlier, such as post-weaning. Therefore, our novel balloon will be helpful to explore the possible mechanism of MS-induced visceral hypersensitivity in early life, contributing to new treatment for IBS.

Colorectal distention is a well-documented method to assist in assessing visceral hypersensitivity (Larsson et al., 2003; Christianson and Gebhart, 2007; Annahazi et al., 2009), and different diameters and materials of the balloon were used for rats and mice (Million et al., 2006; Grabauskas et al., 2020). However, the diameters of the balloon are too large to be used for post-weaning mice, whose body weight are around 10–13 g (Figure 4). Furthermore, the distention pressure of the balloon cannot be monitored in real time (Million et al., 2006; Grabauskas et al., 2020). Thus, we develop a mini-balloon of 3 mm diameter. More importantly, the pressure of our balloon could be monitored in real time (Figure 2). In addition, our balloon is also characterized by the simplicity of the process, flexibility of distention, and the cheap cost, as it was made of latex glove and Teflon tubes (Figure 2). Moreover, using the novel balloon, both AWR threshold and AWR scores at different pressures can be measured. Therefore, our novel balloon may be more accurate and sensitive to evaluate visceral sensitivity compared with reported balloon (Million et al., 2006; Grabauskas et al., 2020), as our novel balloon can measure visceral sensitivity at low and a series of pressures (Figures 5, 6). We found that compared with NS, MS resulted in visceral hypersensitivity as measured by CRD using the novel distention balloon (Figures 5A–D, 6). The result was consistent with a previous study in a rat model of MS, which demonstrated that MS induced visceral hypersensitivity from childhood to adulthood (Yi et al., 2017).

We paid attention to the impact of MS on body weight of the mice. On the one hand, we aimed to investigate whether MS might lead to poor weight gain in MS pups. On the other hand, a significant difference in body weight may have an impact on response to CRD with the same size balloon thereby influencing the results of CRD. We found that MS did not affect the body weight of MS pups as measured at PND 2–25 (Figure 4). Our results indicated that the MS protocol used in this study did not affect body weight, which was consistent with the results of the previous studies (George et al., 2010; Gracia-Rubio et al., 2016). However, one study indicated that MS pups got lower weight during the neonatal period in rats, and various factors have been reported to be associated with the weight of MS pups, such as breastfeeding time, mother’s touching, and caloric expenditure to keep warm (Yi et al., 2017). Inconsistent results may attribute to different species, increased care behavior of dams in mice after neonatal separation, and the efforts we paid to maintain warm ambient temperature.

Our study has some limitations. For example, we did not check the influence of MS on gastrointestinal parameters in post-weaning mice and also did not evaluate whether MS-induced visceral hypersensitivity at post-weaning mice persisted into adulthood. Also, we did not explore the underlying mechanisms in the visceral hypersensitivity observed in the results.

In conclusion, maternal separation induced visceral hypersensitivity in post-weaning mice, which was evaluated via a novel and small size distention balloon in this study. The development of the distention balloon will be helpful to understand the mechanism of pathogenesis of IBS from early life to adulthood.

## Data Availability Statement

The original contributions presented in the study are included in the article/supplementary material, further inquiries can be directed to the corresponding author.

## Ethics Statement

The animal study was reviewed and approved by the Zhejiang University Ethics Committee for Animal Research.

## Author Contributions

ET and MJ designed the present study and critically revised the manuscript for important intellectual content. ET and GL performed the experiments. ET, GL, and TY drafted the manuscript. TY, BC, RG, DY, and MF analyzed the data. All authors have read and approved the manuscript.

## Conflict of Interest

The authors declare that the research was conducted in the absence of any commercial or financial relationships that could be construed as a potential conflict of interest.

## Publisher’s Note

All claims expressed in this article are solely those of the authors and do not necessarily represent those of their affiliated organizations, or those of the publisher, the editors and the reviewers. Any product that may be evaluated in this article, or claim that may be made by its manufacturer, is not guaranteed or endorsed by the publisher.
